# Supplementary material for: Evolutionary dynamics in gut-colonizing Candida glabrata during caspofungin therapy: Emergence of clinically important mutations in sphingolipid biosynthesis
Source: PLoS Pathog. 2024 Sep 9;20(9):e1012521. doi: 10.1371/journal.ppat.1012521 (PMC11412501; doi:10.1371/journal.ppat.1012521)
Supplement: Attachment — Submitted filename: Response to reviewers.pdf [file ppat.1012521.s009.pdf]

We would like to thank the reviewers for a thoughtful assessment of our revised manuscript and constructive feedback. Based on this feedback, we performed additional experiments, including several important controls. We also performed a mass spectrometry experiment to measure the interaction of unlabeled caspofungin with fungal cell membranes. This approach showed that caspofungin association with membranes is greatly reduced in the *fen1Δ* mutant relative to the WT strain, supporting the conclusion reached by imaging cells with Alexa Fluor-labeled caspofungin that the reduced sensitivity of the *fen1Δ* mutant to caspofungin is due at least in part to reduced drug binding to plasma membrane. We also removed the two panels showing results obtained with HaloTag-Fks1 because it became apparent to us that the issue of glucan synthase biogenesis and localization is still too poorly understood and too complex, thus being beyond the scope of the present manuscript. We elaborate on this further in our responses to individual reviewers below.

Reviewer #1:

(1) Figure 3C. Firstly, the results clearly demonstrate that the activity of CSF was reduced by approximately 120-fold (MIC of 0.11 μM for CSF vs. 13 μm for CSF-AF647 against WT) after conjugation with AF647, indicating a significant change in the structure of CSF and loss of activity. Therefore, in contrast to the authors' statement that 'it retained significant antifungal activity' (Line 201), the results show that the conjugate retained very low activity. Secondly, the CSF-AF647 is 98.67% pure. Is the observed low activity of CSF-AF647 due to the presence of a small amount of free CSF molecules or other active intermediates in the sample, i.e., ~1%? Are the 1% impurity and 100-fold decrease of activity a coincidence? Thirdly, there appears to be an issue with the assay of CSF-AF647 activity, as there is no clear concentration dependence in the growth gradient, in contrast to the CSF assay result that shows good gradients. If labeling reduces the activity of a molecule, it is not suitable for studying the molecule's interaction with a cellular target.

We agree with the majority of these comments. Thus, we used mass spectrometry (MS) to ask whether the CSF-AF647 preparation contained a small amount of unconjugated CSF. As we describe in the revised manuscript, this experiment showed that CSF-AF647 contains 0.6% of unlabeled CSF. As the MIC of CSF-AF657 is about 0.85% of that of unlabeled CSF (revised Figure S1B), this means that ~2/3 of that activity might be due to the unlabeled drug, meaning that CSF-AF647 is largely inactive. Whereas we do not fully agree that this makes it completely unsuitable for binding studies (see our response to point 2 below), we acknowledge that we cannot form conclusions based solely on the results obtained with this molecule. Thus, we performed an additional MS-based experiment directly testing whether the interaction of <u>unlabeled CSF</u> with cellular membranes is altered in the *fen1Δ* mutant. The results were unequivocal and fully consistent with those obtained using CSF-AF647: we observed a ~50% decrease in CSF interaction with cellular membranes in the *fen1Δ* relative to the WT strain (revised Figure 3C). Thus, the conclusion that CSF interaction with the plasma membrane is greatly reduced in the *fen1Δ* mutant is supported by two independent, complementary approaches using both labeled and unlabeled CSF.

(2) In Figure 3D (CSF-AF647), firstly, the control using AF647 alone is missing. Secondly, a competition experiment should be conducted by adding different concentrations of unlabeled CSF to see whether it reduces the signal of CSF-AF647 on the cell surface in a concentration-dependent manner.

We also agreed with these suggestions. Unfortunately, the competition experiment was complicated by the fact that unlabeled CSF is extremely potent against *C. glabrata* at very low doses, so including it into the labeling reaction rapidly killed the cells and made them indiscriminately dye-permeable. Thus, this experiment was not pursued further. However, using AF647 alone was very informative. As we describe in the revised manuscript, we purchased AF647 NHS ester, the compound used to label the free amine of CSF, quenched the reactive ester group, incubated it with *C. glabrata* cells, and imaged them using the ONi Nanoimager. Although AF647 retained blinking capacity, it did not bind to *C. glabrata* cells at all, in contrast to CSF-AF647 (revised Figure S1C). Based on this result, we conclude that the interaction between CSF-AF647 and the cell surface is fully due to the CSF moiety and not to AF647. Thus, although the CSF-AF647 has lost the ability to inhibit glucan synthase (probably due to the bulky AF647 adduct, see revised Figure 3A), its interaction with the plasma membrane is driven by the CSF moiety and likely reflects that of unlabeled CSF.

Secondly, while Figure 3A shows that HT-FKS1 is functional, which was tested at one SCF concentration, the JF540 staining patterns are very different between the WT and the fen1Δ strain. There are many more bright dots in fen1Δ cells compared to WT cells, and these dots do not seem localized to the plasma membrane where Fsk proteins are expected to be (There is no colocalization between HT-Fks1 and CSF-F647). The data suggest possible defects in either protein folding or transportation, although a fraction of HT-Fks1 can still confer resistance to CSF. Also, how is Fks2's cellular localization affected in the fen1Δ mutant?

The observation of significant cytoplasmic localization of HT-Fks1 was initially a surprise to us as well. However, a search of the literature showed that cytoplasmic localization with some puncta was observed in multiple instances with fluorescently-labeled Fks1 in different yeasts, including *Saccharomyces cerevisiae* (Guo et al. 2009, PMID: 19564916, see Figure 4C) and *Schizosaccharomyces pombe* (Davidson et al. 2016, PMID: 27898700, see Figures 3 and 5). It was also observed for *C. glabrata* YFP-Fks1 in a recent study done by our collaborators (https://www.biorxiv.org/content/10.1101/2024.05.29.596243v1.full, see Figure 4). Thus, published results from different labs do not align with the expectation that Fks1 should be localized only to the plasma membrane, and the significance of the cytoplasmic puncta, or clusters, is unclear. Given these gaps in knowledge, we realized that the questions of glucan synthase biogenesis, intracellular trafficking, and localization, and how each of these factors is affected by *fen1Δ*, deserve separate focus and are beyond the scope of this manuscript. We are building additional tools for those studies, including both N- and C-terminally fluorescently tagged Fks1 and Fks2.

These issues complicate the interpretation of the results, and the data are not sufficient to conclude that the observed reduced association of CSF-AF647 with the cell surface is responsible for the CSF resistance of the fen1Δ mutant. It is merely a correlation.
We (and other reviewers) disagree that all we show is a correlation. As pointed out by Reviewer #4 below, we also show that deletion of *YPC1* restores both CSF sensitivity and CSF binding in the *fen1Δ* mutant, strongly suggesting that the reduced binding is responsible for the reduced sensitivity. Fully "proving" that this is the case would require extensive medicinal chemistry and fluorescent labeling of CSF analogs and other echinocandins, which is beyond the scope of this manuscript.

(3) Lines 265-270. SNPs in FKS1 HS1 did not affect the fitness of C. glabrata in the gut of mice treated with CSF for two weeks, while mutations in FKS2 increased the fitness. Does Fks1 and Fks2 respond differently to CSF? Why? A relevant question is why Fks2 cellular localization was not examined in Figure 3?
For reasons that are not fully clear, mutations in *FKS2* are much more frequently associated with clinical echinocandin resistance than mutations in *FKS1* (e.g., see PMID: 30895218, PMID: 19546367, PMID: 20837754, PMID: 24126582, etc.), suggesting that mutations in Fks2 hot-spot regions more strongly improve *C. glabrata* fitness in the host during treatment. This is consistent with the results obtained in our mouse model. As we mention above, we are currently analyzing the cellular localization of fluorescently labeled Fks2 as well as Fks1, but the focus of those experiments is on the biogenesis, trafficking, and localization of glucan synthase and is therefore beyond the scope of the current manuscript.

Reviewer #3:
As this manuscript has undergone initial review by three additional reviewers I will keep my assessment brief. This is a well-written manuscript that describes important experiments assessing the in vivo evolution of caspofungin resistance in a murine GI colonization model. These experiments implicate mutations in FEN1 as contributing to the resistant phenotype. Importantly, while much is known about the effect of loss of FEN1 function on echinocandin susceptibility (as pointed out by reviewer 1), this work shows that such loss of function through development of mutations in this gene can be selected for in vivo under caspofungin pressure. This is a key finding. I believe the authors have thoroughly responded to the concerns of reviewer 1 (as well as the other reviewers) and I am supportive of this paper and its importance for the two reasons outlined by reviewer 3. I would also add that the authors then used their experimental observations to explore publicly available WGS data for echinocandin resistant C. glabrata isolates, identified similar mutations in FEN1, and experimentally confirmed that they influence echinocandin susceptibility. This is an important and impactful capstone to this paper in my opinion. I have no additional critique or concerns.
We thank the reviewer for their thoughtful appreciation of both our approach and the importance of our study.

Reviewer #4:
This revised manuscript describes experiments that examine the genotypes and phenotypes behind the emergence of caspofungin resistance in gut colonizing Candida glabrata. This is then related to mutations detected in clinical isolates. As earlier reviewer comments state the importance of sphingolipid synthesis pathway and antifungal resistance has been previously elucidated. Here the study goes a step further to unpick the mechanism to explain why sphingolipid levels, in particular phytosphingosine, influence drug susceptibility. The manuscript is substantially strengthened by the inclusion of new tools and the application of high resolution microscopy to analyse the binding of fluorescently-tagged caspofungin to the plasma membrane in gut-evolved and fen1 mutated strains. The

microscopy clearly demonstrate that there is less caspofungin binding when PHS levels are increased. The authors include a neat control, the ypc1 mutant, which lowers the PHS levels thus increasing caspofungin binding.
We thank the reviewer for their acknowledgement of the improvements in the manuscript and the appreciation of our methodology.

This association between caspofungin binding and PHS levels is clear. What is less clear is how these changes in the membrane lipids alter Fks protein abundance at the plasma membrane and/or Fks enzyme activity leading to changes in cell wall glucan levels. The use of the Halo-tagged Fks1 shows clear differences in Fks1 levels and potentially localization between WT and fen1 mutant. In Figure 3D are the zoomed in panels at the bottom overlays of JF549 and AF647 stains? If so would you have expected to see some overlap in their localisation?
The questions of how changes in membrane lipids alter Fks protein abundance, activity, and inhibition by caspofungin are very interesting but fully beyond the scope of the current manuscript, whose major focus is on the evolution of caspofungin resistance in *C. glabrata* colonizing the host gut, the rapid and frequent appearance of *fen1* mutations, their effect on the interaction between caspofungin and the fungal plasma membrane, the role of phytosphingosine accumulation (*YPC1*), and the identification of loss-of-function *fen1* mutations in clinical *C. glabrata* isolates. For these reasons, and, as mentioned above, because glucan synthase biogenesis and localization are still poorly studied and the published results do not align with the expectation of plasma membrane localization, we have removed the HT-Fks1 panels from Figure 3. We hope that the ongoing and future studies in our labs and others will shed light on these important questions.

The finding that is hard to reconcile is that fen1 mutant doesn't confer caspofungin resistance in the bloodstream immunocompromised mouse model where kidney fungal burdens were reduced as much as WT cells when mice were treated with range of caspofungin doses (up to 5mg/kg). In the discussion the authors suggest that this may be due to concentrations of bioavailable caspofungin in bloodstream versus the gut. Yet FEN1 mutations do emerge in clinical isolates so there is selection pressure to evolve these mutations in potentially diverse host niches (difficult to ascertain this without detailed information on the clinical isolates). This will require further studies to dissect the impact of host niche on the importance of fen1 mutations.
We agree that these are interesting and important questions but that without being able to trace the history of the clinical isolates with *fen1* mutations we cannot answer them at present. We are very interested in the impact of the host niche on the type of mutations that arise and are continuing those studies using mouse models of infection and colonization.

Minor comments:
Line 123, in summary rather than sum
Fixed.

Line 126 states intracellular levels of PHS, do you mean membrane levels?
Yes, fixed.

Line 148, specify Candida species
Fixed.

Line 155 ergosterol
Fixed.

Line 209 Figure 3D
No longer applicable.

Figure 2A it is unconventional to use the term sterilization, you explain its meaning in the legend but as you are monitoring fungal counts in faeces you cannot be completely sure that no fungi remain in the GI tract. Why not state caspofungin treatment or plus caspofungin?
We agree, and we no longer use the term "sterilization", instead stating the fungal burdens were either maintained or depleted during CSF treatment.
