# Supplementary material for: Evolutionary dynamics in gut-colonizing Candida glabrata during caspofungin therapy: Emergence of clinically important mutations in sphingolipid biosynthesis
Source: PLoS Pathog. 2024 Sep 9;20(9):e1012521. doi: 10.1371/journal.ppat.1012521 (PMC11412501; doi:10.1371/journal.ppat.1012521)
Supplement: Attachment — Submitted filename: Hassoun et al Response to Reviewers.docx [file ppat.1012521.s008.docx]

We thank all reviewers for their thoughtful comments. Below please find our point-by-point responses in purple font.

**Reviewer 1:**

First, various aspects of the main findings presented in this manuscript have been reported in other fungi including S. cerevisiae, Aspergilus, C. albicans, and C. glabrata.

Previous findings include:
1.  Disruption of FEN1 reduces cellular sphingolipid levels and results in the accumulation of the long chain base, phytosphingosine. (JBC 272: 17376; 1997).

2.  Sharma S, et al. (2014) Sphingolipid biosynthetic pathway genes FEN1 and SUR4 modulate amphotericin B resistance. Antimicrob Agents Chemother 58(4):2409-14 (In budding yeast).

3. Increased resistance to caspofungin was first reported in C. glabrata fen1Δ by Healey et al. more than 10 years ago, which was attributed to increased cellular levels of some sphingolipid biosynthesis intermediates, including phytosphingosine (Mol Microbiol 86:303; 20120). Similar results were also reported for Candida albicans fen1Δ fen12Δ mutant and Aspergillus nidulans (basA mutant) in another paper by Healey et al. (Antimicrob Agents Chemother 59:3377; 2015). While the authors cited these papers, they failed to explicitly describe the previous findings in the Introduction.

4. Furthermore, a recent paper by Gao et al. demonstrated significant increase in the cellular levels of sphingolipid biosynthesis intermediates in Candida albicans fen1Δ fen12Δ mutants (Chew et al. Nat Commun 9:4495; 2019), significantly reducing susceptibility to fluconazole, and deleting the transcription factor that promote key genes in the synthesis pathways abolished the resistant phenotype.

We are aware of those important studies and cite them in our manuscript. What they show is that deletions of various fungal genes involved in sphingolipid biosynthesis, including *FEN1*, alter antifungal drug sensitivity *in vitro* and that sphingolipid biosynthesis mutants accumulate phytosphingosine. However, those results are not what is novel or significant in our study. As stated by Reviewer 3, the novelty and significance of our study are that: “…it gets at the question of factors that contribute to the emergence of FKS2 mutations and caspofungin resistance specifically as only such mouse experiments could. Secondly, it underscores the need to understand more than just classical genetic determinants of antifungal resistance, but also those genetic determinants of more subtle changes in susceptibility that might represent stepping stones for the emergence of fulminant resistance.” (underscore ours) Indeed, our study is the first to use the clinically relevant mouse gut colonization model to show that caspofungin treatment spurs the dynamic evolution of both drug target and non-drug target mutations in the gut reservoir of *C. glabrata*, that non-drug target mutations, such as *fen1*, provide a fitness benefit to *C. glabrata* in the gut during caspofungin treatment, and that loss-of-function *fen1* mutations also arise in clinical *C. glabrata* isolates. Furthermore, although it has been shown that *fen1* and other sphingolipid biosynthesis mutants accumulate phytosphingosine, our study is the first to demonstrate that, conversely, genetic depletion of phytosphingosine sensitizes *C. glabrata* to caspofungin. Finally, in the revised version we include an additional series of imaging experiments indicating that in the *fen1∆* mutant caspofungin binding to the fungal cell surface is significantly reduced, that this binding is restored in the *fen1∆* *ypc1∆* mutant, and that these changes correlate with the observed decrease and increase in caspofungin sensitivity of these mutants, respectively. These observations suggest that the molecular basis for reduced caspofungin sensitivity in sphingolipid pathway mutants lies in their altered plasma membrane composition, which reduces the interaction with caspofungin. In sum, those are the key novel aspects of our work, and there is nothing in the published literature that shows the host-based effects observed in our study.

Second, the experiments investigating the evolution dynamics was not well-designed.

This opinion is in direct disagreement with both Reviewers 2 and 3. Reviewer 2 specifically commented that “The dynamic analysis of caspofungin treatment on Cg resistance in a mouse exposed to caspofungin using amplicon sequencing is well done”, and Reviewer 3 was wholly positive about our approach, as quoted above, and also stated that “Methods are clearly presented and appropriate in my opinion.” We discuss this issue further in our response to Reviewer 1’s Major Issue #1 below.

**Major issues:**

1. The manuscript extensively discusses the evolution dynamics of CSF-resistant mutants based on the results shown in Figure 4. However, the small sample sizes, i.e., two colonies per day and a total of six mice, are not sufficient to produce any credible evolution patterns. The detection of various mutations in the population on different days appears random, raising more questions than answers. For example, while panel A shows that all fen1 mutations appeared before fks2 mutations, panel B shows the opposite. There is no evidence that the earlier appearance of tolerance or resistance due to fen1 mutations has allowed time for FKS2 resistance to evolve. Although mutations were detected in both fen1 and fks2 in the two colonies from mouse #1 on day 15, the fen1 mutation differs from the one detected on day 9. The fen1-D166N mutant in mouse #2 must be extremely dominant, as it was detected in both colonies from day 7 to day 15; however, no mutations were detected
in FKS2. In mouse #4, the two fen1 mutations detected on day 7 have nothing to do with the FKS2 mutations detected from day 9 to day 15. The fen1-Q10stop mutant found on day 7 in the two colonies from mouse #5 is intriguing. The evolved stop codon at the tenth position of the amino acid sequence makes it almost like the fen∆ mutant, which was shown to completely outcompete the WT strain under CSF treatment (Figure 2B). Yet, the fen1-Q10stop mutation was not detected again from day 7 to day 15. While one can argue that unknown mutations can influence the competitiveness of different mutants, significantly larger sample sizes are required to reveal credible evolutionary patterns of various drug-resistant mutations.

We believe that these comments are entirely about the analysis of individual colonies in Figure 4A. However, as we further elucidate and clarify in the revised version (lines 241-256), this analysis was done *post hoc* on the limited number of colonies collected and stocked in our freezer collection. Thus, this experiment was not designed to reveal evolutionary dynamics but to test the conclusions derived from whole genome sequencing that different *fen1* mutations arise in the mouse gut during caspofungin treatment. Indeed, this experiment confirmed that a variety of *fen1* mutations appear during caspofungin treatment and showed that these mutations arise soon after the start of treatment. Based on this experiment and to get at the evolutionary dynamics, we designed the amplicon sequencing experiment, which was appreciated by both Reviewers 2 and 3, as mentioned above. Finally, the reviewer states that “while panel A shows that all fen1 mutations appeared before fks2 mutations, panel B shows the opposite.” This is not the case, as in no case did an *fks2* mutation appear before an *fen1* mutation. Perhaps the reviewer is talking about mouse 1, where only a *fks2* mutation appeared and no *fen1* was ever observed? We have modified the text to make these distinctions clearer (lines 270-272).

2. In Figure 2C, it is not clear why only the strain FEN1-W145stop (correct nomenclature should be fen1-W145stop) was used in this experiment but not fen1∆. These two mutants are not equal because fen1-W145stop carries several other mutations which may have unknown influence on this strain’s fitness. The result shows that the fen1-W145stop mutant exhibits similar sensitivity to CSF as the WT strain during systemic infection in mice. However, only one CSF concentration, 5 mg/kg, was used in this experiment. This is a very high concentration compared with the dose used in humans (50-70 mg per day for adults; assuming the average weight of a person is 60 kg). It is likely that this concentration is significantly higher than the MIC of both strains, masking the difference in susceptibility to CSF between them. The experiment should be repeated using lower CSF concentrations, such as 1 mg / kg. Also, is there an estimate of the CSF concentration in the gut where CSF is IP-administered at 20 mg / kg?

These are all valid concerns, and we have addressed them by repeating the mouse systemic infection experiments using the *fen1∆* strain and a range of caspofungin concentrations: 1.25, 2.5, and 5 mg/kg. As described in the revised manuscript, the results are consistent with those reported in our original manuscript, in that *fen1∆* did not alter kidney fungal burdens after systemic infection and caspofungin treatment (lines 176-183, Figure 2C). GI concentrations of caspofungin after treatment with 20mg/kg can be found in Healey at al. AAC 2017 (PMID: 28971865). We discuss the implications of reduced GI caspofungin concentrations relative to those in the plasma/organs in the Discussion paragraph beginning on line 371.

3. The authors used different gut-evolved fen mutants in different experiments. For example, they used GI-240 to generate the data in Figure 2, but GI-299 and GI-240 for Figure 3 and Figure 5, respectively. This raises the question whether different strains exhibit significantly different phenotypes, particularly fitness in the gut under CSF treatment. This is worrisome because many of the evolved strains carry multiple mutations.

In every case the gut-evolved strains behaved similarly to the *fen1∆* mutant, and we have not observed any evidence that “different strains exhibit significantly different phenotypes”, although of course it is theoretically possible. In light of this concern, all of the additional experiments for the revision were done using the *fen1∆* strain.

4. Lines 188-189. It is described that ‘one non-fen1 gut-evolved strain (GI-63)’ was included in the lipidomics analysis. However, the strain was not seen in Figure 3A.

We apologize for this typo – instead of GI-63 the text should say GI-113. This has been fixed in the revision.

**Minor issues:**

1. Figure 1. Column 2: What does ‘genotype’ refer to? I thought all the GI strains are evolved strains and most of them carry mutations. Thus, what does WT mean? Also, the four gene deletion mutants were not explained, and they are not mentioned anywhere else in the text.

These points have been clarified in the revised legend to Figure 1 as follows: “All parental strains used to colonize the mice belonged to the DPL1021 (ATCC90030) background. Some of the parental strains carried the gene deletions shown in the “Genotype” column; “WT” indicates absence of such gene deletions. These gene deletions were made as part of our investigation of the roles of the cell wall integrity pathway (*slt2∆* and *yps1∆*, (40)) and error-prone DNA replication (*rev1∆* and *rev3∆*) in the evolution of caspofungin-resistant mutants in gut-colonizing *C. glabrata*.”

2. Figure 2A. I figured that sterilization and no sterilization mean if mice were treated with the antibiotic or not. If so, it’s better to use + or – antibiotic treatment. The use of a single antibiotic cannot sterilize the gut of mice. Antibiotic cocktails are often used but still cannot ensure sterilization.

As is stated in the materials and methods, all mice were treated with antibiotics prior to fungal colonization, and we use an antibiotic cocktail (piperacillin-tazobactam). As we clarify in the revised legend to Figure 2A, “Sterilization refers to *C. glabrata* gut levels decreasing to undetectable levels during caspofungin treatment and not rebounding during the timeframe of the experiment.” Thus, it is a measure of caspofungin efficacy in eliminating gut-colonizing *C. glabrata*.

3. Figure 2A. The near zero CFU on day 7 in three mice inoculated with WT C. glabrata are strange. Excluding these outliers, gut colonization of the WT strain would not be significantly different from that of the mutants.

We are not sure what this is referring to. In figure 2A, on day 7 several mice had fungal burdens below the limit of detection, but *C. glabrata* cells were still present in the gut, as the burdens rebounded on day 9 and afterward. This happens sometimes during treatment due to evolution of resistance, and though it doesn’t happen very often, we would not call this behavior as “outliers”.

4. Line 160. Nomenclature: is W145* the same as W145stop? If so, please use W145* and explain it when it first appears.

We now clarify in the legend to Figure 1 that * signifies a stop codon and refer to this mutant as W145* throughout the manuscript.

5. Line 399, italicize C. glabrata.

This was fixed in revision.

6. Line 400, citation format for Healey et al. is different from the rest.

This was fixed in revision.

**Reviewer 2**

This is nice work and succinctly presented. The dynamic analysis of caspofungin treatment on Cg resistance in a mouse exposed to caspofungin using amplicon sequencing is well done.

We appreciate the reviewer’s positive comments.

**Major issues**

If insight could be provided into the basis of the effect of sphingolipid deficiency on echinocandin resistance, then the impact of this work would be enhanced. For example, is the endocytosis of the Fks proteins influenced by defects in normal sphingolipid production? Is the activity of the Fks proteins enhanced or the ability of echinocandins to inhibit these enzymes reduced?

In response to this concern and that of reviewer 1, in the revised version we include the results of high-resolution imaging experiments (Figures 3, 4C, and Supplementary Figure 1B), which show that in the *fen1∆* mutant caspofungin binding to *C. glabrata* cells is significantly reduced, that this binding is restored in the *fen1∆* *ypc1∆* mutant, and that these changes correlate with the observed decrease and increase in caspofungin sensitivity of these mutants, respectively (lines 185-218 and 233-239 of Results and lines 387-407 of Discussion). These observations suggest that the molecular basis for reduced caspofungin sensitivity in sphingolipid pathway mutants lies in their altered plasma membrane composition – notably, the increased phytosphingosine levels – which reduces the caspofungin-membrane interaction and therefore also reduces caspofungin-mediated inhibition of membrane-embedded glucan synthase. We have also begun to test the hypothesis that glucan synthase trafficking or localization are altered by the *fen1∆* mutation by tagging the Fks1 protein with the HaloTag epitope and analyzing its localization in the WT and *fen1∆* strains using high resolution imaging. These results are likewise presented and discussed in the lines mentioned above.

**Minor issues**

None.

**Reviewer 3**

This paper is significant in my opinion for two reasons. First, it gets at the question of factors that contribute to the emergence of FKS2 mutations and caspofungin resistance specifically as only such mouse experiments could. Secondly, it underscores the need to understand more than just classical genetic determinants of antifungal resistance, but also those genetic determinants of more subtle changes in susceptibility that might represent stepping stones for the emergence of fulminant resistance. Such information might ultimately lead to strategies to mitigate the emergence of resistance.

The introduction is clear and concise with the needed background, the results are clearly presented with good use of figures and tables, and the discussion is a sound treatment of the findings. Methods are clearly presented and appropriate in my opinion.

We appreciate the reviewer’s positive comments.

**Major issues**

None.

**Minor issues**

1. Aside from the original evolved strains, I didn’t see any MICs for other echinocandins. One would expect no change or increased susceptibility to micafungin and anidulafungin in the mutants. Is this observed and does this represent a therapeutically exploitable phenotype?

Revised Figures 1A, 4B and 6A show the MIC data for caspofungin, micafungin, and anidulafungin for all strains. Micafungin MICs are indeed reduced and we discuss the potential exploitability of this observation in the Discussion (lines 417-422).

2. Additional discussion of the clinical relevance would be of value in my opinion. While this is interesting and informative biology, there may be ways in which this information might inform how echinocandins might be used to reduce the emergence of resistance.

The implications of our results for clinical evolution of resistance are discussed in the Discussion paragraphs starting from lines 341 (present in the original submission) and 408 (highly revised).
